# *In Vivo* Targeting of Clostridioides difficile Using Phage-Delivered CRISPR-Cas3 Antimicrobials

**DOI:** 10.1128/mBio.00019-20

**Published:** 2020-03-10

**Authors:** Kurt Selle, Joshua R. Fletcher, Hannah Tuson, Daniel S. Schmitt, Lana McMillan, Gowrinarayani S. Vridhambal, Alissa J. Rivera, Stephanie A. Montgomery, Louis-Charles Fortier, Rodolphe Barrangou, Casey M. Theriot, David G. Ousterout

**Affiliations:** a Locus Biosciences, Inc., Morrisville, North Carolina, USA; b Department of Population Health and Pathobiology, College of Veterinary Medicine, North Carolina State University, Raleigh, North Carolina, USA; c Department of Pathology and Laboratory Medicine, Lineberger Comprehensive Cancer Center, University of North Carolina School of Medicine, Chapel Hill, North Carolina, USA; d Department of Microbiology and Infectious Diseases, Faculty of Medicine and Health Sciences, Université de Sherbrooke, Sherbrooke, Canada; e Department of Food, Bioprocessing and Nutrition Sciences, North Carolina State University, Raleigh, North Carolina, USA; University of Oklahoma Health Sciences Center

**Keywords:** *Clostridioides difficile*, CRISPR-Cas, Cas3, phage, lysogeny, CRISPR

## Abstract

Clostridioides difficile is an important nosocomial pathogen that causes approximately 500,000 cases of C. difficile infection (CDI) and 29,000 deaths annually in the United States. Antibiotic use is a major risk factor for CDI because broad-spectrum antimicrobials disrupt the indigenous gut microbiota, decreasing colonization resistance against C. difficile. Vancomycin is the standard of care for the treatment of CDI, likely contributing to the high recurrence rates due to the continued disruption of the gut microbiota. Thus, there is an urgent need for the development of novel therapeutics that can prevent and treat CDI and precisely target the pathogen without disrupting the gut microbiota. Here, we show that the endogenous type I-B CRISPR-Cas system in C. difficile can be repurposed as an antimicrobial agent by the expression of a self-targeting CRISPR that redirects endogenous CRISPR-Cas3 activity against the bacterial chromosome. We demonstrate that a recombinant bacteriophage expressing bacterial genome-targeting CRISPR RNAs is significantly more effective than its wild-type parent bacteriophage at killing C. difficile both *in vitro* and in a mouse model of CDI. We also report that conversion of the phage from temperate to obligately lytic is feasible and contributes to the therapeutic suitability of intrinsic C. difficile phages, despite the specific challenges encountered in the disease phenotypes of phage-treated animals. Our findings suggest that phage-delivered programmable CRISPR therapeutics have the potential to leverage the specificity and apparent safety of phage therapies and improve their potency and reliability for eradicating specific bacterial species within complex communities, offering a novel mechanism to treat pathogenic and/or multidrug-resistant organisms.

## Abstract (precis)

Clostridioides difficile is a bacterial pathogen responsible for significant morbidity and mortality across the globe. Current therapies based on broad-spectrum antibiotics have some clinical success, but approximately 30% of patients have relapses, presumably due to the continued perturbation to the gut microbiota. Here, we show that phages can be engineered with type I CRISPR-Cas systems and modified to reduce lysogeny and to enable the specific and efficient targeting and killing of C. difficile *in vitro* and *in vivo.* Additional genetic engineering to disrupt phage modulation of toxin expression by lysogeny or other mechanisms would be required to advance a CRISPR-enhanced phage antimicrobial for C. difficile toward clinical application. These findings provide evidence into how phage can be combined with CRISPR-based targeting to develop novel therapies and modulate microbiomes associated with health and disease.

## INTRODUCTION

Bacteriophages are viruses infecting prokaryotes that have been studied for over a century as potential antimicrobials that feature exquisite host specificity. Phage therapy offers the advantage of narrow-spectrum activity for the treatment of Clostridioides difficile infection (CDI) (1–3), potentially reducing or eliminating the alterations to the gut microbiota that contribute to the recurrence of CDI after treatment (4–6). Preclinical studies of phage therapies for CDI have demonstrated C. difficile-specific activity in complex bioreactor models and efficacy in *in vivo* models, highlighting the feasibility of this approach in the treatment of CDI (7–9). However, all C. difficile phages isolated and characterized to date are considered temperate, a lifestyle feature involving the incorporation of a prophage into the bacterial genome, thereby providing phage resistance to the lysogenic host via repressor-mediated immunity or superinfection exclusion (10). Since no obligately lytic phages have been discovered for C. difficile, we aim to use synthetic biology approaches to reduce or remove the impact of lysogeny on the therapeutic efficacy of a single phage for this organism.

Modern synthetic biology techniques and genome-editing technologies have facilitated the development of engineered phages to increase their efficacy by introducing other desirable properties, such as expanded host range, biofilm degradation, elimination of lysogeny, and addition of genes to arm phages with secondary antimicrobial payloads (11–15). CRISPR-Cas systems have been identified to be a potential payload that can be delivered by phages and used to directly and specifically kill the organism of interest in a sequence-specific manner via targeting of the chromosome or to eliminate plasmids carrying undesirable pathogenesis or antibiotic resistance genes (16, 17). CRISPR-Cas systems function as sequence-specific adaptive immune systems in bacteria through the activity of RNA-guided DNA nucleases, where a single-stranded RNA molecule complexes with Cas proteins and then recognizes a DNA target through RNA-DNA base pairing, as well as recognition of a short adjacent DNA sequence (a protospacer-adjacent motif [PAM]) by Cas proteins (18, 19). Type I CRISPR-Cas systems include a family of proteins that share the signature Cas3 nuclease, which creates a single-strand nick at the defined DNA sequence, followed by processive exonucleolytic degradation of the targeted strand (19–21). In contrast to other CRISPR-Cas systems, CRISPR-Cas3 targeting of chromosomal DNA results in robust bacterial death regardless of the gene targeted and does not have apparent strain- or sequence-dependent activity (16, 21). Moreover, CRISPR-Cas3 is the predominant system widely distributed in prokaryotes and accounts for nearly 60% of all identified systems to date (19). Type I-B CRISPR-Cas systems are found in nearly all sequenced isolates of C. difficile and have been shown to mediate interference against exogenously introduced DNA (22, 23).

We hypothesized that the type I-B CRISPR-Cas system in C. difficile could be exploited as an antimicrobial via phage delivery of a lethal genome-targeting CRISPR array. Such a strategy would be expected to improve phage efficacy during lytic replication and reduce lysogeny by intolerance of the bacterial genome-targeting CRISPR from the nascent prophage.

## RESULTS

### CRISPR phage engineering and mechanism of action.

The C. difficile genome-targeting CRISPR was constructed using the native leader and consensus CRISPR from the highly expressed endogenous CR11 array in C. difficile 630 (23). We selected 236 nucleotides (nt) of the native leader sequence, which drives expression of the CRISPR array from canonical σ70 and RNA polymerase recognition motifs. The repeat sequence was generated by deriving the 29-nt consensus of 15 repeats from the C. difficile 630 CR11 array. The optimal length of the spacer sequence was defined by the length distribution of all spacers in ∼220 queried genomes, which was determined to be 37 nucleotides; thus, the spacer sequence was constrained to 37 nucleotides downstream of the consensus PAM sequence (5′-CCW-3′) and was selected for its high conservation across C. difficile strains (22, 23). The CRISPR expression construct was then validated by cloning into pMTL84151 and subsequent conjugation into model C. difficile strains R20291 and 630, which have high conjugation efficiencies and which have been demonstrated to have endogenous type I-B activity (23). Successful genome targeting by the CRISPR was defined by the reduction in the conjugation efficiency of pMTL84151::CRISPR compared to that of the pMTL84151 vector control. The CRISPR plasmid exhibited an approximately 1-log reduction in conjugation efficiency (see Fig. S1a in the supplemental material). Thus, the genome-targeting CRISPR caused a significant loss in viable transconjugants, implying lethal DNA degradation caused by the endogenous type I-B system in C. difficile and validating its use for improving the efficacy of bacteriophage-mediated bacterial suppression.

10.1128/mBio.00019-20.1 FIG S1 Conjugation efficiency of the type I-B Clostridium difficile genome-targeting CRISPR array. (a) The crRNA was cloned into pMTL84151 and conjugated into model C. difficile strains 630 and R20291. Viable transconjugants of the crRNA plasmid were recovered at approximately a 1-log lower frequency than those of the empty pMTL84151 plasmid control. (b) Transmission electron micrographs of wild-type and CRISPR phage variants. Comparison of the wild-type phage and crPhage morphology shows no differences in capsid size or sheath length. (c) Dependency of the reduction in the numbers of CFU on the input MOI of phage variants. An MOI of ≥1 favors a rapid decrease in the numbers of CFU but results in a rebound by 24 h. In contrast, an MOI of ≤0.01 results in moderate reductions in the numbers of CFU that continue to decrease up to 24 h. For panels a and c, the data are presented as the mean ± standard error of the mean. The significance in panel a was determined by the *t* test by the Holm-Sidak method. *, *P* < 0.05; **, *P* < 0.01; ***, *P* < 0.001; ****. *P* < 0.0001. Download FIG S1, PDF file, 1.2 MB. Copyright © 2020 Selle et al. 2020 Selle et al. This content is distributed under the terms of the Creative Commons Attribution 4.0 International license.

**FIG 1:**
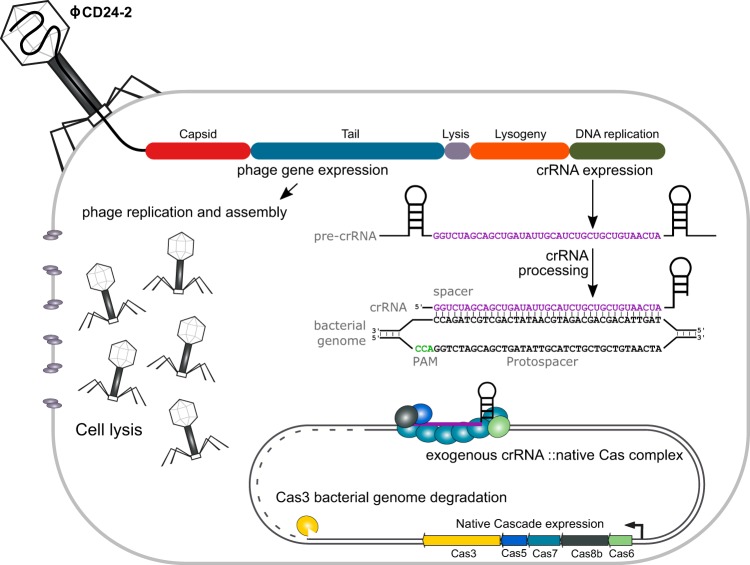
Overview of CRISPR phage engineering and mechanism of action. The genome of phage ϕCD24-2 was modified to encode a bacterial genome-targeting CRISPR array composed of a repeat-spacer-repeat meeting the requirements of the conserved C. difficile type I-B system. The genome-targeting CRISPR array is transduced into the bacterial cell during infection and is expressed concurrently with the lytic genes of the bacteriophage. Cell death occurs by two independent mechanisms of action: irreparable genome damage by the natively expressed type I-B Cas effector proteins directed by the CRISPR RNA and cell lysis by the holin and endolysin expressed during lytic replication.

We next sought to demonstrate that phage-mediated delivery of a genome-targeting CRISPR could enhance wild-type phage activity to more efficiently kill a target population of C. difficile. Given the efficacy of the genome-targeting CRISPR, we moved the leader-driven repeat-spacer-repeat construct onto the genome of the C. difficile bacteriophage ϕCD24-2 (the CRISPR-enhanced phage is referred to as crPhage from here on) (Fig. 1). Since it was unclear whether genetic modification of the phage would affect its morphology, we compared the phage morphology of wild-type ϕCD24-2 (referred to as wtPhage from here on) with that of the crPhage by negatively stained transmission electron microscopy TEM (Fig. S1b). Both the wtPhage and crPhage displayed typical *Myoviridae* morphology and were consistent with a previous report on ϕCD24-2 (24). In terms of host range, wtPhage was capable of infecting 10/87 strains, including a few strains from ribotypes 001, UM3, and UM11 from a clinical isolate strain panel, as determined by the spot plating assay (25) (Table S1). The crPhage infected the same 10/87 strains as the wtPhage, evidence that insertion of the CRISPR into the phage genome did not affect the morphology or host range (data not shown). We also assessed the titers achieved by routine amplification and storage stability of the crPhage compared to those for the wtPhage and found no differences between them over the course of 4 weeks at 4°C (data not shown).

10.1128/mBio.00019-20.4 TABLE S1 Wild-type phage ϕCD24-2 host range. Download Table S1, XLSX file, 0.01 MB. Copyright © 2020 Selle et al. 2020 Selle et al. This content is distributed under the terms of the Creative Commons Attribution 4.0 International license.

### *In vitro* activity against C. difficile and lysogeny formation of wild-type and engineered phages.

**FIG 2:**
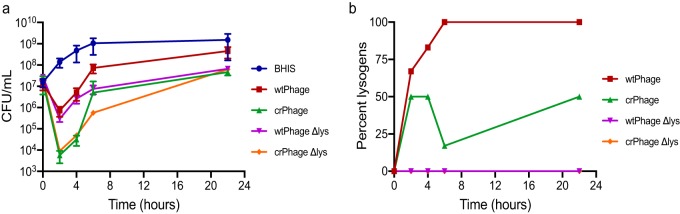
*In vitro* comparison of the activity and lysogen formation rates of wild-type and engineered phage variants. (a) Time course of the reduction in the numbers of CFU during *in vitro* infection by bacteriophage at an input MOI of 0.1. CRISPR-engineered phage offers a clear improvement in the reduction in the numbers of CFU between 2 and 6 h, but by 24 h, all phage-treated cultures recover. Interestingly, there was no observable effect of lysogeny gene knockout on the activity of the phage. Data are presented as the mean ± standard error of the mean. BHIS, brain heart infusion supplemented with 0.1% taurocholate. (b) Time course of PCR-based detection of lysogeny in surviving bacterial colonies after phage infection. The CRISPR-enhanced phage exhibits impaired lysogen formation. Phage variants lacking key lysogeny genes exhibit no detectable lysogeny *in vitro*. Two biological replicates were performed per treatment per experiment.

We next performed *in vitro* CFU reduction experiments against C. difficile strain CD19, which is a host that is susceptible to ϕCD24-2 infection by plaque assay and which is capable of colonizing mice and producing toxin *in vivo* (24). The cultures treated with wtPhage exhibited an approximately 1-log reduction in the numbers of CFU after 2 h of incubation but recovered rapidly. In contrast, the crPhage both increased the depth of the maximum reduction of the numbers of CFU after 2 h of infection (approximately 3 logs) and decreased the recovery of the culture over 24 h (Fig. 2a). The multiplicity of infection (MOI) used for treatment also played a role in the observed depth of killing and outgrowth suppression: an MOI of ≥1 favored rapid bacterial killing with a higher log reduction, whereas MOIs of 0.01 or 0.001 suppressed C. difficile better specifically at the 24-h time point (Fig. S1c). These CFU rebound data are consistent with the occurrence of bacteriophage-insensitive mutants, the development of which may be promoted at high initial MOIs (29). Specifically, the prevalence of lysogens, which are insensitive to reinfection, increased over the time course of the experiment (Fig. 2a and b).

### Activity of wild-type and engineered phages in a CDI mouse model.

**FIG 3:**
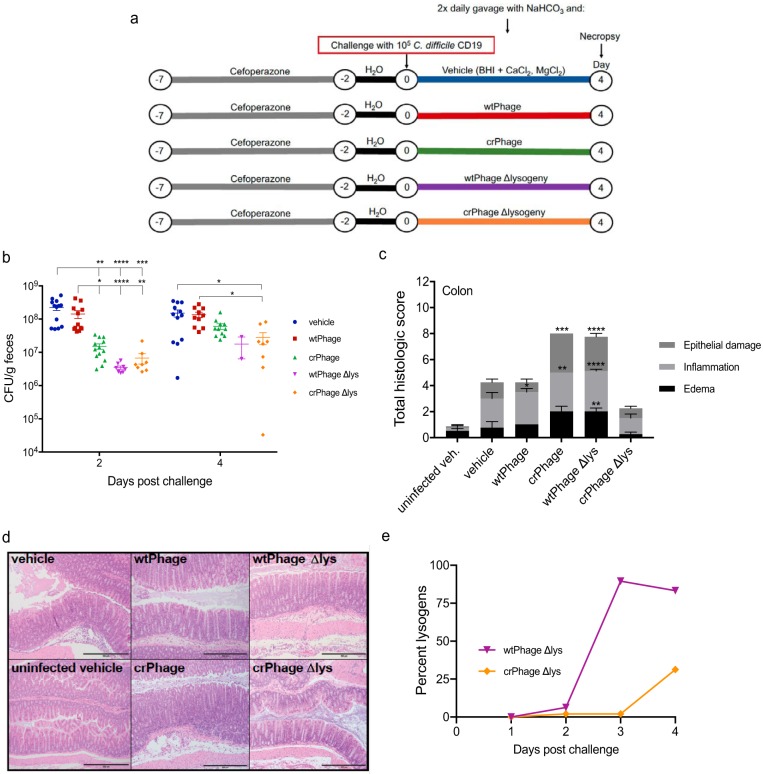
A bacteriophage encoding a CRISPR targeting the C. difficile genome reduces the C. difficile burden and clinical signs of disease *in vivo*. (a) Schematic depicting the experimental design, timeline, and treatment groups. For the vehicle-, wtPhage-, and crPhage-treated groups, 20 mice were used in each group over three experiments; for the wtPhage Δlys- and crPhage Δlys-treated groups, 8 mice were used in each group. (b) Numbers of fecal C. difficile vegetative CFU from mice in each treatment group at days 2 and 4 postchallenge. For day 2, the vehicle-treated group contained 13 mice, the wtPhage-treated group contained 12 mice, the crPhage-treated group contained 13 mice, and the wtPhage Δlys- and crPhage Δlys-treated groups contained 8 mice each. For day 4, the vehicle-treated group contained 12 mice, the wtPhage-treated group contained 10 mice, the wtPhage Δlys-treated group contained 2 mice (6/8 mice treated with the wtPhage Δlys failed to defecate after three handling attempts), and the crPhage Δlys-treated group contained 8 mice. (c) Total histological scores of colons harvested at day 4 postchallenge. An uninfected, vehicle-treated group was included to control for the effects of the vehicle on colonic tissue. The uninfected vehicle-, wtPhage Δlys-, and crPhage Δlys-treated groups contained 8 mice each; the vehicle-, wtPhage-, and crPhage groups-treated contained 4 mice each. Each variable was scored on a scale of from 0 to 4; thus, the total summary score for maximum tissue damage is 12. (d) Representative images of hematoxylin- and eosin-stained colonic tissue from day 4 postchallenge for which the results are shown in panel c. Bars, 500 μm. (e) Percentage of lysogens isolated from the feces of mice treated with either wtPhage Δlys or crPhage Δlys over the course of the experiment (*n* = 8 mice per day, with 6 colonies per mouse being screened by PCR). For panels b and c, data are presented as the mean ± standard error of the mean. *P* values were determined by the Kruskal-Wallis one-way ANOVA with Dunn’s correction for multiple comparisons for panels b and c. *, *P* < 0.05; **, *P* < 0.01; ***, *P* < 0.001; ****, *P* < 0.0001.

Given the increased efficacy of the crPhage in killing C. difficile *in vitro*, we sought to determine how treatment with the crPhage would affect the outcome of disease in a mouse model of CDI *in vivo*. Initially, we defined the kinetics of infection with C. difficile strain CD19. Mice were given the antibiotic cefoperazone in their drinking water for 5 days to make them susceptible to CDI (27) and then were challenged with C. difficile CD19 spores on day 0 (Fig. S2a). At 4 days postchallenge, we observed significant weight loss, high cecal burdens of C. difficile CD19, and significant histopathological changes to the cecum, including edema, inflammation, and epithelial damage (Fig. S2b and d). For these reasons, we chose 4 days postchallenge as the endpoint for the experimental phage therapy model. As mentioned above, mice were given cefoperazone in their water, and at 4 h after spore challenge, the mice were given via oral gavage 100 μl of 6% (wt/vol) NaHCO_3_ in water to increase the pH of the stomach and protect the administered phages from degradation during transit through the stomach. The mice then received one of three treatments via oral gavage: vehicle, wtPhage, or crPhage (Fig. 3a). Mice treated with the crPhage (green) had a significantly reduced fecal C. difficile burden, with an approximately 10-fold reduction in the numbers of vegetative C. difficile CFU at 2 days postchallenge relative to the numbers in mice given either the vehicle alone (blue) or the wtPhage (red) (*P* = 0.0013 and *P* = 0.0232) (Fig. 3b). In contrast, mice treated with the wtPhage had vegetative cell CFU in their feces, similar to the findings for mice treated with vehicle, suggesting that the wtPhage is not as virulent *in vivo*. By day 4, the numbers of vegetative cell CFU rebounded in mice treated with the crPhage, though the numbers of CFU were still lower than those from vehicle- and wtPhage-treated mice. At the time of necropsy on day 4, the cecal CFU also showed a significant reduction in the C. difficile vegetative cell burden in the crPhage-treated group compared to that in mice treated with wtPhage (*P* = 0.0175) (Fig. S3b). Given our *in vitro* observations, we hypothesized that the rebound in the numbers of CFU at day 4 was due to lysogeny, although additional phage resistance mechanisms, including the receptor-mediated resistance commonly seen in monophage treatments, may have also played a role (28).

10.1128/mBio.00019-20.2 FIG S2 C. difficile CD19 colonizes and causes disease in cefoperazone-treated mice. (a) Schematic depicting the experimental design and timeline. Data are for 16 mice. (b) Change in weight over time in mice challenged with C. difficile CD19. (c) Numbers of total CFU and the number of spore CFU in the cecal contents from infected mice at days 4 and 9 postchallenge (*n* = 8 mice per day). (d) Total histological score for cecal tissue from uninfected and infected mice from day 4 postchallenge (*n* = 8 mice per day). For panels b and c, data are presented as the mean ± standard error of the mean. *P* values were determined by the Kruskal-Wallis one-way ANOVA with Dunn’s correction for multiple comparisons for panel b and a two-tailed Mann-Whitney test for panel d. *, *P* < 0.05; **, *P* < 0.01; ***, *P* < 0.001; ****, *P* < 0.0001. Download FIG S2, PDF file, 0.08 MB. Copyright © 2020 Selle et al. 2020 Selle et al. This content is distributed under the terms of the Creative Commons Attribution 4.0 International license.

10.1128/mBio.00019-20.3 FIG S3 A CRISPR-enhanced bacteriophage reduces the C. difficile burden and clinical signs of disease *in vivo.* (a) Change in weight in mice from each treatment group at 4 days postchallenge. The uninfected vehicle-treated group contained 4 mice; the vehicle-, wtPhage-, and crPhage-treated groups contained 14 mice each, and the wtPhage Δlys- and crPhage Δlys-treated groups contained 8 mice each. (b) Numbers of vegetative C. difficile CFU from cecal contents harvested at 4 days postchallenge. The vehicle- and wtPhage-treated groups contained 10 mice each, the crPhage-treated group contained 9 mice, and the wtPhage Δlys- and crPhage Δlys-treated groups contained 8 mice each. (c) Total histological score for cecal tissue harvested at 4 days postchallenge. Each variable has a scale of from 0 to 4, so the maximal histological summary score is 12. The uninfected vehicle-, wtPhage Δlys-, and crPhage Δlys-treated groups contained 8 mice each, and the vehicle-, wtPhage-, and crPhage-treated groups contained 4 mice each. (d) Representative images of cecal tissue harvested from mice of each treatment group on day 4 postchallenge. Bars, 500 μm. (e) Numbers of PFU from fecal contents at 2 and 4 days postchallenge. For all treatments, 8 to 12 fecal samples were tested, except for the wtPhage Δlys treatment (*n* = 2) at day 4 due to a lack of defecation. For panels a, b, c, and e, data are presented as the mean ± standard error of the mean. *P* values were determined by the Kruskal-Wallis one-way ANOVA with Dunn’s correction for multiple comparisons for panels a, b, c, and e. *, *P* < 0.05; **, *P* < 0.01; ***, *P* < 0.001; ****, *P* < 0.0001. Download FIG S3, PDF file, 0.9 MB. Copyright © 2020 Selle et al. 2020 Selle et al. This content is distributed under the terms of the Creative Commons Attribution 4.0 International license.

Six individual C. difficile colonies from the feces of each mouse were screened by PCR at 2 and 4 days postchallenge, resulting in a total of 96 and 120 colonies screened per wtPhage and crPhage treatment group, respectively. The smaller number of colonies screened from the wtPhage treatment group reflects the fact that several mice from that group did not produce fecal samples. On day 2, we found that 81% of the colonies screened from mice treated with wtPhage contained lysogens (data not shown). In contrast, on day 2, only 10% of the colonies from mice treated with the crPhage contained lysogens. By day 4, 62% of the colonies screened from mice treated with wtPhage and 73% of the colonies from crPhage-treated mice were lysogens. The lysogens were confirmed by PCR to have the correct identity; that is, lysogens from groups treated with wtPhage contained the wild-type phage, and lysogens from groups treated with crPhage were recombinant (data not shown). However, upon sequencing 35 crPhage lysogens from day 4, we found that 30 of them had lost the spacer and one repeat from the CRISPR region. Four failed to produce good sequence data, and one maintained the spacer and both repeats. This observation may indicate that lysogens with an intact genome-targeting CRISPR are likely to be unstable and select for excision of the CRISPR spacer, given that the excision of spacers by repeat-mediated recombination is a primary form of escape from genome and plasmid targeting (16).

### Improved activity against C. difficile *in vivo* with lysogeny mutant phages.

Given the pervasive lysogeny by day 4, we constructed mutant wtPhage and crPhage lacking key lysogeny genes (wtPhage Δlys and crPhage Δlys, respectively). We performed CFU reduction assays, as before, and over the course of 22 h, we did not detect lysogens from cultures treated with either phage *in vitro* (Fig. 2). We then repeated the *in vivo* mouse model of CDI and gave mice either phage as treatment. Mice treated with the wtPhage Δlys had a nearly 2-log reduction in their fecal C. difficile burdens at 2 days postchallenge relative to the mice given vehicle or wtPhage alone (*P* < 0.0001 for both) (Fig. 3b). By day 2, mice given crPhage Δlys had a nearly 4-fold reduction in the number of fecal CFU relative to that in mice treated with crPhage alone and a nearly 2-log reduction compared to that in vehicle-treated mice (Fig. 3b; Fig. S3c and d). By day 4, the numbers of fecal CFU had increased in all treatment groups, though the mice given crPhage Δlys still had significantly lower numbers of fecal CFU than the mice given vehicle or those treated with the wtPhage (*P* = 0.0472 and *P* = 0.0125) (Fig. 3b). The C. difficile burdens in the day 4 cecal content from mice treated with either wtPhage Δlys or crPhage Δlys were not significantly different from those in mice given vehicle or from those in mice given the parent phage treatment (Fig. S3b). Significant histopathological changes were detected in the ceca (Fig. S3c and d) and colons (Fig. 3c and d) from mice treated with the crPhage and wtPhage Δlys; however, the cecal and colonic tissues, as well as the weight loss, in mice treated with crPhage Δlys were not significantly different from those in uninfected, vehicle-treated control mice (Fig. 3c and d and Fig. S3a, c, and d). This finding suggests that the activity of the modified recombinant phage is associated with a lower level of tissue damage in the host.

Although we did not detect lysogeny from wtPhage Δlys and crPhage Δlys *in vitro*, curiously, we were able to detect lysogens in the feces of mice treated with each, albeit at a lower frequency with the latter phage (Fig. 3e). PCR screening confirmed that the detected lysogens were not the result of contamination with wtPhage or crPhage. Interestingly, unlike the crPhage lysogens, the crPhage Δlys lysogens maintained the spacer and both repeats. At this time, it is unclear whether other C. difficile genome-encoded prophage genes are able to functionally complement those genes removed from the wtPhage Δlys and crPhage Δlys genomes. It has been previously reported that lysogeny affects the bacterial physiology of C. difficile. When lysogenized by ϕCD38-2, the R20291 strain of C. difficile exhibited increased toxin gene expression *in vitro*, suggesting that the increased tissue pathology that we observed in mice treated with crPhage and wtPhage Δlys may be due to a similar phenomenon (30). It is also increasingly clear that prophages can have significant impacts on many facets of the biology of C. difficile, so it is possible that some of the observed effects on host tissues could be due to toxin-independent physiological changes in the lysogens as well (30). Collectively, these results highlight the need to elucidate the mechanisms of lysogeny and its impacts on host cell physiology to further the development of crPhage therapies from temperate phages. Specifically, additional genetic engineering to disrupt the phage modulation of toxin expression by lysogeny or other mechanisms would be required to advance a crPhage antimicrobial for C. difficile toward clinical application.

## DISCUSSION

Two seminal studies have previously demonstrated the activity of phagemid-delivered CRISPR-Cas9 in selectively targeting pathogenic bacteria in mixed populations and infection models (26, 31). The work presented here represents a significant advancement in the field of phage-delivered CRISPR antimicrobials. Notably, we have shown that an endogenous type I CRISPR-Cas system can be exploited as a potent antimicrobial by phage delivery of a bacterial genome-targeting CRISPR with activity that surpasses lytic phage activity alone. While this approach could theoretically result in CRISPR-mediated resistance to the crPhage, previous work has inferred that the C. difficile CRISPR system does not readily acquire new spacers, as indicated by the lack of differentiating spacers across multiple strains (22). Moreover, the typical frequency at which spacers are acquired even in highly active systems approaches 1 in 10^7^ bacteria, which is expected to be lower than the frequency at which single phage resistance is acquired by receptor modification (32). Finally, CRISPR resistance can be expected to occur during infection of hosts with functional CRISPR-Cas systems, and thus, expression of genome-targeting CRISPR RNAs (crRNAs) from the phage genome may ensure that clonal populations of phage-resistant clones are unable to survive and propagate. Additionally, inclusion of a genome-targeting spacer in a recombinant phage genome is expected to be an orthogonal method of eliminating lysogeny in temperate phages, may reduce the risk of the unexpected restoration of prophage genes from homologous prophages in the host, and may enable killing of prophage-containing hosts displaying superinfection immunity.

We have shown that a C. difficile temperate phage genome can be readily modified, including by addition of desirable payloads and alteration of genes related to lysogeny. By altering temperate phages to promote lytic activity, an additive antimicrobial effect between lysis and bacterial genome degradation by the type I-B CRISPR-Cas system is achieved, resulting in improved reductions in the bacterial load *in vitro* and *in vivo*. The development of lysogeny in surviving clones of C. difficile also indicates a high rate of phage infection *in vivo* by oral delivery. Interestingly, the crPhage Δlys culture recovered over the course of the experiment, implying that a phage resistance mechanism distinct from lysogeny arose.

Resistance development against single phage treatments is common and occurs by various mechanisms documented elsewhere. Although the use of a single phage in this study was suitable for comparing genetically engineered derivatives for their activity and efficacy against a single bacterial host, there are significant limitations associated with using a single phage in therapeutic settings. Specifically, the host range is minimal, and any single mechanism of resistance will abrogate the activity of the phage. In order to address both the host range and the efficacy limitations of a single phage, multiple phages with orthogonal host binding receptors are typically used to cover more strains and reduce the impact of the generation of resistance against any single phage.

Future improvements to this approach would include the development of crRNA arrays targeting multiple genes with repeat structures that reduce the probability of recombination and spacer excision, as well as identifying and removing putative lysogenic genes that promote lysogeny *in vivo*, as noted in this study. Additionally, therapeutic phage cocktails can be readily developed to expand the host range, reduce resistance by using phages with complementary receptors, and exploit the general method of CRISPR and lysogeny knockout engineering demonstrated in this study to improve efficacy and generate therapeutically relevant lytic C. difficile phage. Altogether, we demonstrate the feasibility of engineering a recombinant bacteriophage that expresses a CRISPR targeting the C. difficile genome and that can be modified to reduce and potentially eliminate lysogeny, thereby creating a new class of phage therapeutics for this important nosocomial pathogen. Our data demonstrate the potential for engineered phage therapeutics to reduce the C. difficile burden *in vivo* and reduce disease severity in the complex milieu of the mammalian gastrointestinal environment, highlighting the use of CRISPR-based phage therapeutics as a promising new approach for precision antimicrobials and microbiome modulation. Furthermore, these results open new avenues for the engineering of phages with CRISPR-Cas systems to modulate the composition of microbiomes in health and disease.

## MATERIALS AND METHODS

### Bacterial strains and culture conditions.

All bacterial strains were stored at −80°C in their respective medium supplemented with a final concentration of 15% (vol/vol) glycerol or as spore stocks generated as described previously (33). The C. difficile strains used in this study were provided by Louis-Charles Fortier (34) and Seth Walk (25). Strains were struck from freezer stocks onto brain heart infusion (BHI) agar plates (Teknova) and incubated at 37°C in a Coy anaerobic chamber using 85% nitrogen, 5 to 10% hydrogen, and 5% carbon dioxide. Strains were subcultured by inoculating BHI broth with a single colony and incubating at 37°C. BHI agar was supplemented with cycloserine (8 μg/ml), cefoxitin (25 μg/ml), and thiamphenicol (Tm; 15 μg/ml) to select for recombinant C. difficile. The Escherichia coli strains were streaked onto LB agar plates (Teknova) and incubated at 37°C. The E. coli strains were grown in LB broth, which was supplemented where necessary with carbenicillin (50 μg/ml), chloramphenicol (15 μg/ml), or erythromycin (200 μg/ml).

### DNA isolation and engineering.

All kits and reagents were used according to the manufacturers’ instructions. Plasmid and genomic DNA isolation was performed using a Zymo Research plasmid miniprep kit and a Quick DNA fungal/bacterial miniprep kit, respectively. Phage DNA purification was performed as described previously (29). Restriction enzymes, T4 DNA ligase, and DNA polymerases were from New England Biolabs. Routine PCR was performed using *Taq* DNA polymerase, and high-fidelity amplifications were performed using Phusion DNA polymerase. All PCR products were visualized by gel electrophoresis using 0.8% agarose with GelRed. Recombinant phages containing an expression cassette encoding a bacterial genome-targeting CRISPR RNA and replacing phage gene gp75 by homologous recombination were created. To reduce the potential of ϕCD24-2 to form lysogens, a region of the genome encoding the *c*I repressor and integrase gene was deleted. The bacterial strains and plasmids used in this study are listed in Table S2 in the supplemental material.

10.1128/mBio.00019-20.5 TABLE S2 Bacterial strains and plasmids used in this study. Download Table S2, DOCX file, 0.02 MB. Copyright © 2020 Selle et al. 2020 Selle et al. This content is distributed under the terms of the Creative Commons Attribution 4.0 International license.

### Phage morphology.

Phages were prepared for TEM using a modification of the method described by Fortier and Moineau (35). Prior to observation, 1.5 ml of crude lysate was centrifuged for 1 h at 4°C and 24,000 × *g*. A fraction of the supernatant (approximately 1.4 ml) was gently removed and discarded, and 1 ml of ammonium acetate (0.1 M, pH 7.5) was added to the remaining lysate, which was then centrifuged as described above. This step was performed twice. Washed phage samples were visualized by negative-stain transmission electron microscopy. A glow-discharged Formvar/carbon-coated 400-mesh copper grid (Ted Pella, Inc., Redding, CA) was floated on a 25-μl droplet of the sample suspension for 5 min and transferred quickly to 2 drops of deionized water, followed by transfer to a droplet of 2% aqueous uranyl acetate stain for 30 s. The grid was blotted with filter paper and air dried. Samples were observed using a JEOL JEM-1230 transmission electron microscope operating at 80 kV (JEOL USA, Peabody, MA), and images were taken using a Gatan Orius SC1000 charge-coupled-device camera with Gatan Microscopy (suite 3.0) software (Gatan, Inc., Pleasanton, CA).

### Phage-handling procedures.

ϕCD24-2 in the prophage state was induced from C. difficile CD24 by UV irradiation (302 nm) as described previously (29). Both the wild-type ϕCD24-2 phage (wtPhage) and the CRISPR-enhanced phage (crPhage) were propagated by amplification on C. difficile CD19. An overnight culture of C. difficile CD19 was subcultured 1:100 into BHI broth and incubated at 37°C to an optical density at 600 nm (OD_600_) of 0.10. MgCl_2_ and CaCl_2_ were added to final concentrations of 10 mM and 1 mM, respectively. Bacteriophage was added at a multiplicity of infection (MOI) of 0.02, and the cultures were incubated at 37°C for 7 to 8 h, until some clearance of the culture was observed. Amplification cultures were removed from the anaerobic chamber and centrifuged at 4,000 × *g* for 20 min. The supernatants were filtered through 0.45-μm-pore-size filters. Phages were precipitated with polyethylene glycol (PEG) by adding 0.2 volume of 20% PEG 8000, 2.5 M NaCl solution (catalog no. P4137; Teknova) and incubating overnight at 4°C. On the following day, the phage suspensions were centrifuged for 10 min at 13,000 × *g* at 4°C. The supernatants were decanted, and the pellets were resuspended in 5 ml BHI. The phage suspensions were centrifuged for 10 min at 13,000 × *g* at 22°C to remove the residual PEG. The supernatants were transferred to fresh tubes, and 10 mM MgCl_2_ and 1 mM CaCl_2_ were added. The lysates were stored at 4°C until use. The phage titer was determined by the soft agar overlay method. Briefly, 800 μl of 2 M MgCl_2_, 20 μl of 2 M CaCl_2_, 500 μl of C. difficile CD19 at an OD_600_ of 0.3 to 0.6, and 100 μl of phage at a range of dilutions were added to 3 ml of 0.375% BHI agar (Teknova). The mixture was poured onto a 1.5% BHI agar plate, allowed to solidify, and incubated at 37°C anaerobically overnight. On the following day, the plaques were counted and the number of phages per milliliter was calculated.

### CRISPR targeting sequence.

The following sequence consists of the leader sequence (underlined), the repeat sequences (bolded), and the spacer sequence targeting RNase Y (italicized): GTGCTTTTAAATTTACAAAGTATTCCATTTTAATTTTATAGTTTAGATTTTATGATATAATAAAAATATAGAAGTTTTGCAGTGTGCGATATTTGTTACAAAGTAGGGCTTAATACTTGAAATCTAAGATGTTGAGGGTGCGTGATAAGTGTTATCAATTGCACTATTGCCCGCTCACTGCAATTTTAAGAGTATTGTATATATGTAGGTATTGGAAATGCTAAGTTTATTTTGGG**GTTTTAGATTAACTATATGGAATGTAAAT***GGTCTAGCAGCTGATATTGCATCTGCTGCTGTAACTA***GTTTTAGATTAACTATATGGAATGTAAAT**.

### *In vitro* phage efficacy.

Phages were diluted to a titer of 2.0 × 10^8^ PFU/ml in BHI plus 10 mM MgCl_2_ and 1 mM CaCl_2_. Overnight cultures of C. difficile were subcultured 1:100 into BHI and incubated at 37°C to an OD_600_ of 0.20. Then, 10 mM MgCl_2_ and 1 mM CaCl_2_ were added to the bacterial culture and the culture was mixed 1:1 with phage or BHI plus 10 mM MgCl_2_ and 1 mM CaCl_2_. At 0, 2, 4, 6, and 22 h, 10-fold serial dilutions down to 1:10^6^ were made in BHI. A 5-μl volume of each dilution was spotted onto BHI agar and allowed to dry into the surface of the plate. The plates were incubated overnight at 37°C. On the following day, the number of colonies in the densest countable spot was determined and used to calculate the number of cells per milliliter. This experiment was repeated 4 times with wtPhage and crPhage; 1 time with wtPhage and wtPhage Δlys; and 1 time with wtPhage, crPhage, wtPhage Δlys, and crPhage Δlys.

### Animals and housing.

Male and female C57BL/6 mice (age, 5 weeks old) were purchased from The Jackson Laboratory (Bar Harbor, ME) for use in infection experiments. The food, bedding, and water were autoclaved, and all cage changes were performed in a laminar flow hood. The mice were subjected to a 12-h light and 12-h dark cycle.

Animal experiments were conducted in the Laboratory Animal Facilities located on the North Carolina State University (NCSU) College of Veterinary Medicine (CVM) campus. The animal facilities are equipped with a full-time animal care staff coordinated by the Laboratory Animal Resources (LAR) division at NCSU. The NCSU CVM is accredited by the Association for the Assessment and Accreditation of Laboratory Animal Care International (AAALAC). The Institutional Animal Care and Use Committee (IACUC) at the NCSU CVM approved this study. Trained animal handlers in the facility fed and assessed the status of animals several times per day. Those assessed as moribund were humanely euthanized by CO_2_ asphyxiation.

### C. difficile spore preparation.

Spores of C. difficile strain CD19 were prepared as described by Thanissery et al. (33) and Perez et al. (36). Briefly, 2 ml of an overnight culture of CD19 in Columbia broth was added to 40 ml of Clospore medium and incubated at 37°C for 7 days, after which time the spores were centrifuged and washed 5 times in cold sterile water. Alternatively, 500 μl of an early-log-phase-growth culture of C. difficile was spread onto a 70% SMC–30% BHI agar plate and incubated at 37°C for 3 to 4 days, as described by Edwards and McBride (37). The growth was then scraped off the agar plate and resuspended in 10 ml of sterile phosphate-buffered saline (PBS). Ten milliliters of 96% ethanol was added, and the mixture was vortexed and allowed to sit on the benchtop for 1 h. The spore mix was then centrifuged at 3,000 rpm for 10 min. The pellet was suspended in 10 ml sterile PBS and centrifuged again. The pellet was then suspended in 1 ml sterile PBS and heated to 65°C for 20 min. Spores prepared by either method were enumerated at the time of their preparation and prior to preparation of the inoculum via serial dilution in the anaerobic chamber and plating onto brain heart infusion supplemented with 0.1% taurocholate. Spore inocula were also enumerated immediately prior to *in vivo* challenge. The latter method was found to reliably produce significantly more spores than the previous method, so it was utilized after the first biological replicate of the mouse challenge experiments.

### Antibiotic administration and infection with C. difficile.

The mice (*n* = 80 over three experiments, male and female) were administered 0.5 mg/ml of cefoperazone (dissolved in Gibco distilled water [catalog no. 15230147]) in their drinking water for 5 days to render them susceptible to C. difficile colonization (23). All mice were then given distilled water to drink for 2 days, after which they were orally gavaged with 10^5^ spores of C. difficile strain CD19 in 25 μl (34). Animals were monitored for clinical signs of disease (weight loss, inappetence, wet stool, hunched posture, ruffled fur), and animals were humanely sacrificed via CO_2_ asphyxiation if they met the clinical endpoint of a loss of 20% of their initial body weight. Fecal pellets were collected daily, weighed, passed into the anaerobic chamber, and diluted 1:10 (wt/vol) in sterile anaerobic PBS (catalog no. 10010023; Gibco). The diluted pellets were serially diluted and plated onto the C. difficile selective medium cefoxitin d-cycloserine fructose agar (CCFA) to enumerate the vegetative cells. Necropsy was performed on days 2 and 4 postchallenge. The cecal content was harvested for the enumeration of C. difficile on CCFA. Cecal and colon tissue was harvested for histopathology analysis. Note that it was found that not all mice had defecated when stool was collected for bacterial enumeration, especially mice exhibiting severe clinical signs of disease.

### Treatment with phage.

At approximately 4 h after challenge with C. difficile spores, the mice were orally gavaged with 100 μl of 6% (wt/vol) NaHCO_3_ solution to neutralize stomach acid. After approximately 30 min, this was followed by one of five treatments in 100 μl: vehicle, which was BHI, 10 mM MgCl_2_, and 1 mM CaCl_2_ (CD19, *n* = 20); wtPhage (*n* = 20); recombinant crPhage (*n* = 20), all over the course of three independent biological replicates; wtPhage lysogeny mutant (wtPhage Δlys, *n* = 8); or crPhage lysogeny mutant (*n* = 8, crPhage Δlys) from one biological replicate. One group was given cefoperazone and vehicle but was not challenged with C. difficile spores (*n* = 4 from one biological replicate). This group served as the control for the effects of the vehicle on gut tissue in the histopathological analysis. The treatment gavages were repeated twice daily, approximately 8 to 9 h apart, for the duration of the experiment (Fig. 3a).

### Screening for lysogens.

Resuspended fecal pellets were plated on BHI agar supplemented with cycloserine and cefoxitin. Individual colonies were restreaked onto BHI agar twice to isolate bacteria away from phage particles. Colonies were PCR screened using primers to detect the presence of a phage lysogen. For detecting wild-type lysogens, forward primer TGGTAATAGATAGCCTACATTAGTA and reverse primer GTTTACAATTAAATAGCCACT were used. For detecting crPhage lysogens, forward primer TTACTCTACTTTAAGATTCAATTCA and reverse primer CCCAAAATAAACTTAGCATTTC were used. Colonies positive for prophage presence were further screened using primers specific to the wild-type or CRISPR-engineered phage. For titration of phage in fecal samples, resuspended fecal pellets were centrifuged for 10 min at 4,000 × *g*. Supernatants were filtered through 0.45-μm-pore-size spin filters and used in soft agar overlays as described above.

### Histopathological examination of the mouse cecum and colon.

At the time of necropsy, the cecum and colon were prepared for histology by placing the intact tissue into histology cassettes, which were stored in 10% buffered formalin for 48 h and then transferred to 70% ethyl alcohol for long-term storage. The tissue cassettes were further processed and paraffin embedded and then sectioned. Hematoxylin- and eosin-stained slides were prepared for histopathological examination (University of North Carolina Animal Histopathology & Lab Medicine core). Histological sections were coded, randomized, and scored in a blind manner by a board-certified veterinary pathologist (S.A.M.). Edema, inflammation (cellular infiltration), and epithelial damage for the cecum and colon were each scored on a scale of from 0 to 4, based on a previously published numerical scoring scheme (38). Edema scores were as follows: 0, no edema; 1, mild edema with minimal multifocal submucosal expansion (2 times) or a single focus of moderate submucosal expansion(2 or 3 times); 2, moderate edema with moderate multifocal submucosal expansion (2 or 3 times); 3, severe edema with severe multifocal submucosal expansion (3 times); and 4, the same as a score of 3 but with diffuse submucosal expansion. Cellular infiltration scores were as follows: 0, no inflammation; 1, minimal multifocal neutrophilic inflammation of scattered cells that do not form clusters; 2, moderate multifocal neutrophilic inflammation (greater submucosal involvement); 3, severe multifocal to coalescing neutrophilic inflammation (greater submucosal involvement with or without mural involvement); and 4, the same as a score of 3 but with abscesses or extensive mural involvement. Epithelial damage was scored as follows: 0, no epithelial changes; 1, minimal multifocal superficial epithelial damage (vacuolation, apoptotic figures, villus tip attenuation/necrosis); 2, moderate multifocal superficial epithelial damage (vacuolation, apoptotic figures, villus tip attenuation/necrosis); 3, severe multifocal epithelial damage (same as above) with or without a pseudomembrane (intraluminal neutrophils, sloughed epithelium in a fibrinous matrix); and 4, the same as a score of 3 but with significant pseudomembrane or epithelial ulceration (focal complete loss of epithelium). Photomicrographs were captured on an Olympus BX43 light microscope with a DP27 camera using cellSens Dimension software.

### Statistical analyses.

Statistical tests were performed using Prism (version 7.0b) software for Mac OS X (GraphPad Software, La Jolla, CA, USA). Statistical significance was set at a *P* value of <0.05 for all analyses. For the *in vivo* experiments, a Kruskal-Wallis one-way analysis of variance (ANOVA) followed by Dunn’s multiple-comparisons *post hoc* test was used to calculate significance between treatment groups. A Geisser-Greenhouse two-way ANOVA with Sidak’s multiple-comparisons *post hoc* test was used to calculate significance between *in vitro* toxin expression data. A Student's *t* test corrected for multiple comparisons using the Holm-Sidak method was used to calculate significant differences in conjugation efficiency between groups, and a Mann-Whitney two-tailed *t* test was used to evaluate histological scores in CD19 infection.

### Biological materials availability.

Base shuttle vectors are available commercially through Chain Biotech. E. coli and C. difficile strains and wild-type ϕCD24-2 phage are available through C.M.T. and L.-C.F. The CRISPR plasmid and engineered phages are proprietary and to be released at the sole discretion of Locus Biosciences.

### Data availability.

The data presented in this study are available upon request.
