# Supplementary figures and images for: In Vivo Targeting of Clostridioides difficile Using Phage-Delivered CRISPR-Cas3 Antimicrobials
Source: mBio. 2020 Mar 10;11(2):e00019-20. doi: 10.1128/mBio.00019-20 (PMC7064742; doi:10.1128/mBio.00019-20)

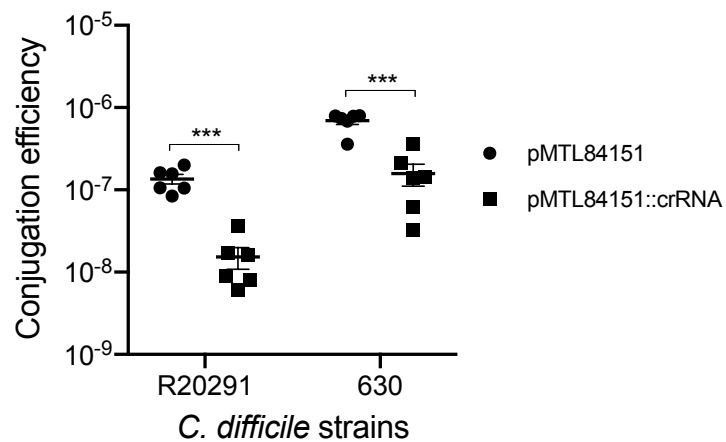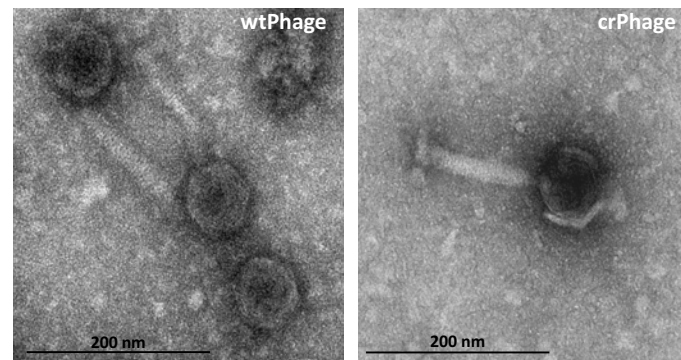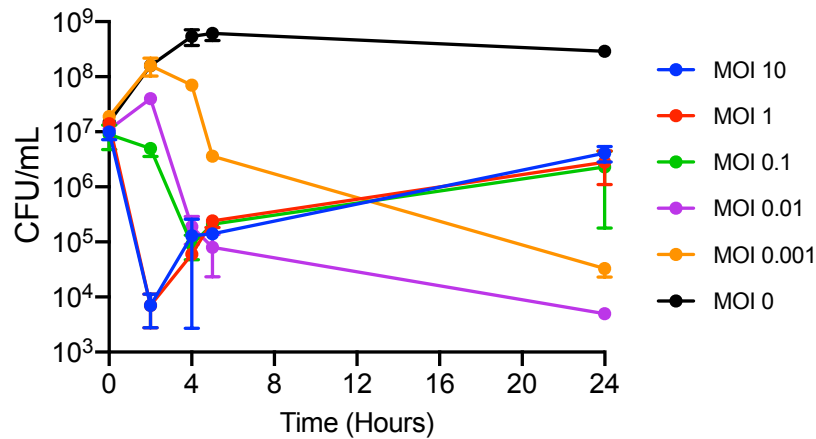

Supplement: FIG S1 [file mBio.00019-20-sf001.pdf]

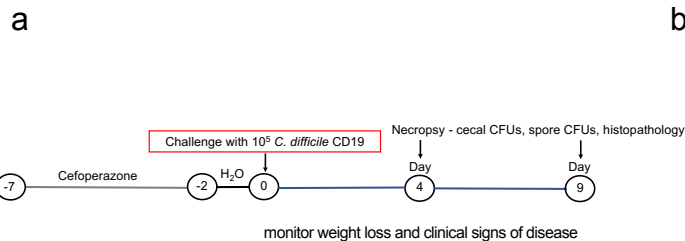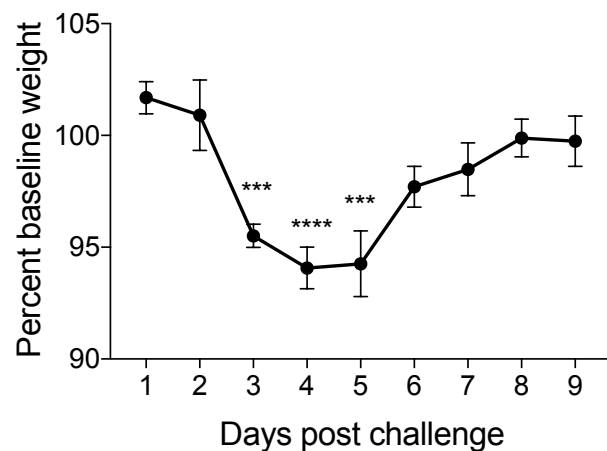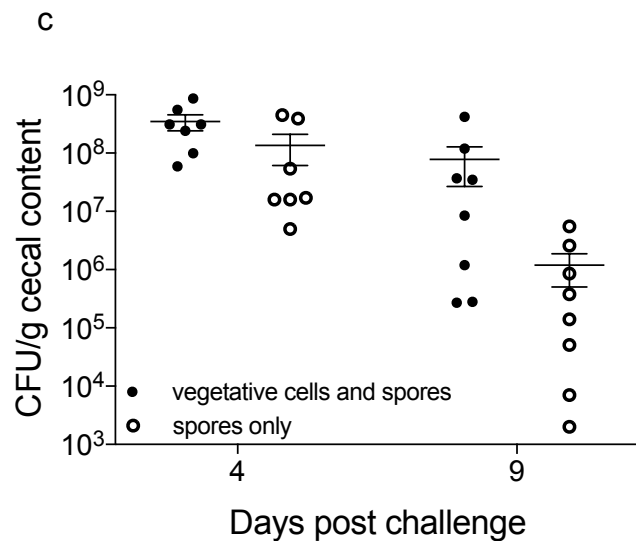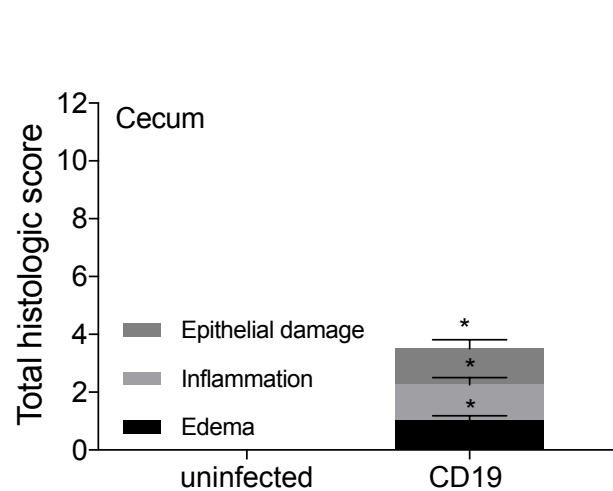

Supplement: FIG S2 [file mBio.00019-20-sf002.pdf]

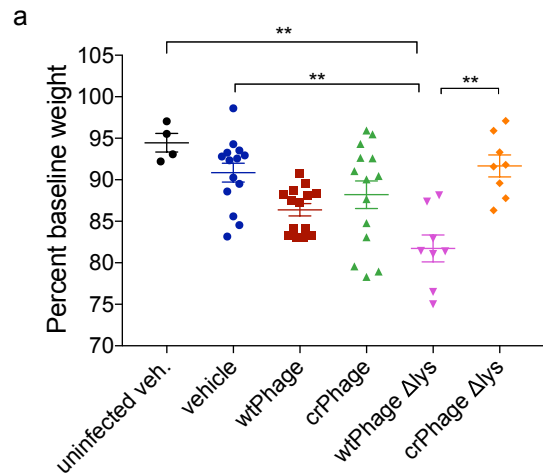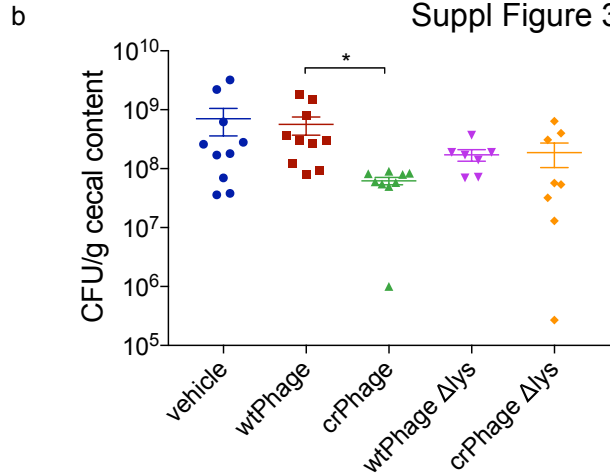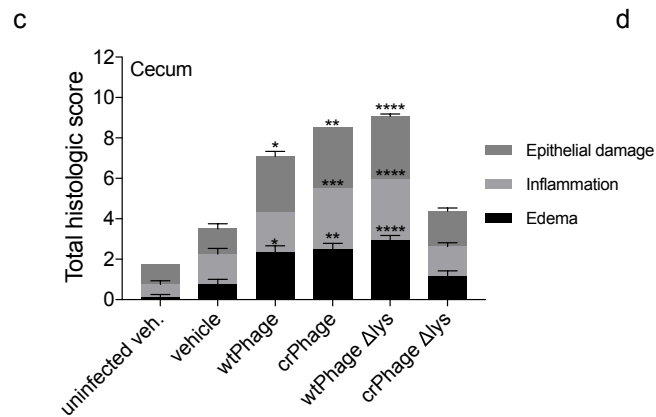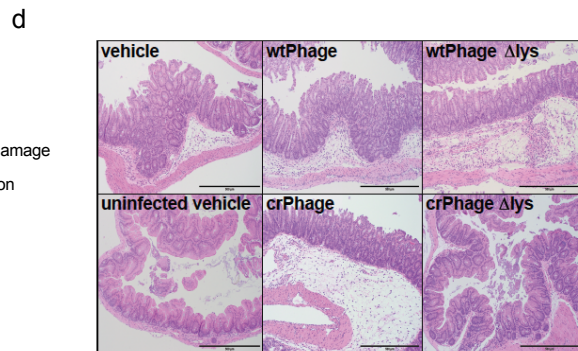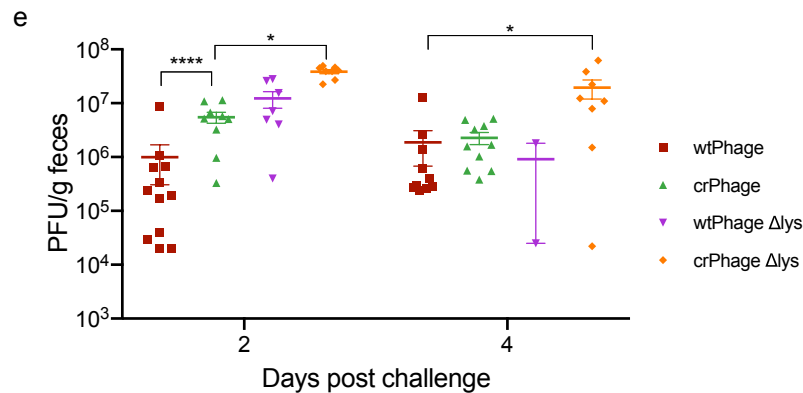

Supplement: FIG S3 [file mBio.00019-20-sf003.pdf]
