# Supplementary material for: In Vivo Targeting of Clostridioides difficile Using Phage-Delivered CRISPR-Cas3 Antimicrobials
Source: mBio. 2020 Mar 10;11(2):e00019-20. doi: 10.1128/mBio.00019-20 (PMC7064742; doi:10.1128/mBio.00019-20)
Supplement: TABLE S2 [file mBio.00019-20-st002.docx]

**Table S2. Bacterial strains and plasmids used in this study.**

| **Species** | **Strain** | **Purpose** | **Source** | **Reference** |
| --- | --- | --- | --- | --- |
| *E. coli* | DH5α | Cloning host | NEB | ^40^ |
| *E. coli* | SD46 | Conjugation donor | Theriot |  |
| *C. difficile* | CD19 | Amplification host; animal model target | Fortier | ^34^ |
| *C. difficile* | CD24 | ϕCD24-2 lysogen | Fortier | ^34^ |

| **Plasmid** | **Purpose** | **Source** | **Reference** |
| --- | --- | --- | --- |
| pMTL84151 | Cloning vector, Conjugation control | Chain Biotech | ^41^ |
| pMTL82151 | Cloning vector | Chain Biotech | ^41^ |
| pMTL82251 | Cloning vector | Chain Biotech | ^41^ |
| pMTL84151::CRISPR | CRISPR validation | Locus | This study |
